# Robotic pancreaticoduodenectomy for pancreatic head cancer and periampullary lesions

**DOI:** 10.1002/ags3.12457

**Published:** 2021-03-28

**Authors:** Yi‐Ming Shyr, Shin‐E Wang, Shih‐Chin Chen, Bor‐Uei Shyr, Bor‐Shiuan Shyr

**Affiliations:** ^1^ Division of General Surgery Department of Surgery Taipei Veterans General Hospital and School of Medicine National Yang Ming University Taipei Taiwan

**Keywords:** cancer, da Vinci Surgical System, pancreatic head, pancreaticoduodenectomy, periampullary

## Abstract

Pancreaticoduodenectomy, so‐called “Whipple operation,” is a time‐consuming and technically demanding complex operation. Traditionally, this procedure has been performed most usually by open approach, which results in a large and painful wound. With the introduction of laparoscopic and robotic surgery, minimally invasive surgery (MIS) has emerged as a worldwide trend to improve wound cosmesis and to minimize wound pain. Although MIS for pancreaticoduodenectomy has also been attempted at some centers, the role of MIS, either robotic or laparoscopic approach, has not been well‐established for complex pancreaticoduodenectomy. Given that laparoscopic pancreaticoduodenectomy has been limited by its technical complexity and the high level of advanced laparoscopic skills required for pancreatic reconstruction, a robotic surgical system is introduced to overcome several limitations related to the laparoscopic approach. Providing high‐quality three‐dimensional (3‐D) vision, high optical magnification, articulation of robotic instruments, greater precision with suture targeting, and elimination of surgeon tremor, robotic surgical systems innovatively perform more delicate and complex procedures involving extensive dissection and suturing techniques such as pancreaticoduodenectomy. Although associated with longer operative time, robotic pancreaticoduodenectomy (RPD) has been claimed to have the benefits of less delayed gastric emptying, less blood loss, shorter length of postoperative stay, and lower wound infection rate, as compared with the traditional open pancreaticoduodenectomy (OPD). Moreover, RPD seems to be not only technically feasible but also justified without compromising the survival outcomes for pancreatic head and ampullary adenocarcinomas. Therefore, RPD could be recommended not only to surgeons but also to patients in terms of surgical feasibility, surgical outcomes, and patient satisfaction.

## Graphical abstract

Robotic approach allows surgeons to perform more complicated and delicate procedures such as pancreaticoduodenectomy involving extensive dissection and restoration of digestive tract continuity for the pancreas, bile duct, and gastrointestinal tract. Robotic pancreaticoduodenectomy has been reported as associated with less blood loss, less delayed gastric emptying, shorter length of postoperative stay, and lower wound infection rate, but longer operative time, as compared with the traditional open pancreaticoduodenectomy.

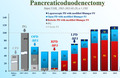

## 1 INTRODUCTION

**FIGURE 1:**
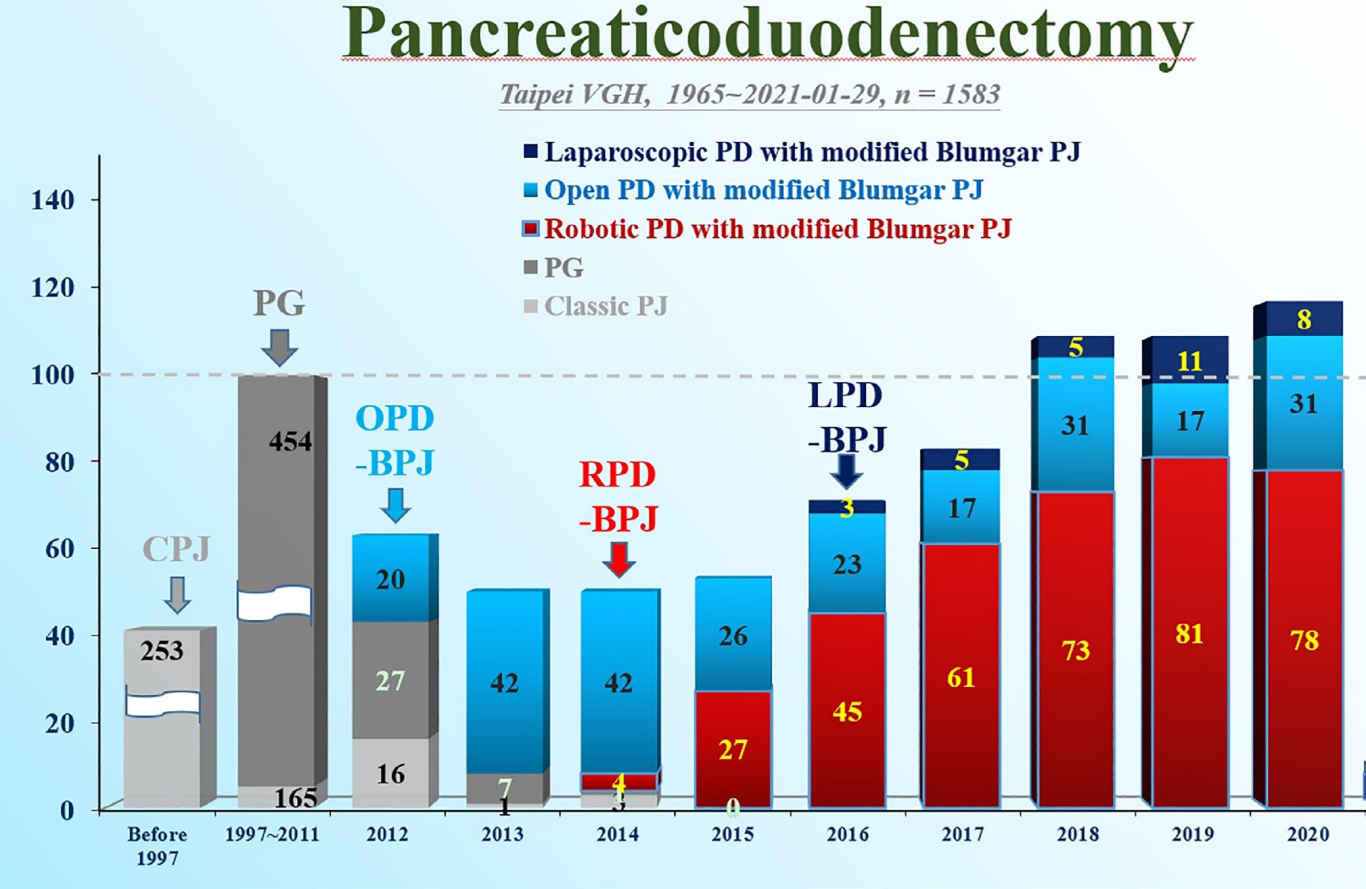
Pancreaticoduodenectomy at Taipei Veterans General Hospital. CPJ, classic pancreaticojejunostomy; PG, pancreaticojejunostomy, OPD‐BPJ, open pancreaticoduodenectomy with modified Blumgart pancreaticojejunostomy; RPD‐BPJ, robotic pancreaticoduodenectomy with modified Blumgart pancreaticojejunostomy; LPD‐BPJ, laparoscopic pancreaticoduodenectomy with modified Blumgart pancreaticojejunostomy

Pancreaticoduodenectomy, so‐called “Whipple operation,” is a time‐consuming and technically demanding complex operation. Traditionally, this procedure has been performed most usually by open approach, which results in a large and painful wound. With the introduction of laparoscopic and robotic surgery, minimally invasive surgery (MIS) has emerged as a worldwide trend with improving wound cosmesis and mitigating wound pain.^1^, ^2^, ^3^, ^4^, ^5^ Although MIS for pancreaticoduodenectomy has also been attempted at some centers, the role of MIS, either by robotic or laparoscopic approach, has not been well‐established for the complex pancreaticoduodenectomy. The pancreas team led by Y. M. Shyr and S. E. Wang at Taipei Veterans General Hospital have been endeavoring to develop robotic pancreaticoduodenectomy (RPD) since 2014.^6^ With the experience of more than 1580 cases of pancreaticoduodenectomy and over 375 cases of RPD (Figure 1), some remarkable results have been achieved in RPD,^7^, ^8^, ^9^ including: (a) shorter hospital stay after RPD, as early as on post‐operative day 6 in five cases of RPD; (b) better cosmesis and smaller wounds, as small as 3 ~ 4 cm by RPD, about 1/10 of the 30 ~ 40 cm wounds by traditional OPD; (c) nearly “no” blood loss in four cases of RPD, with a mean of 120‐150 c.c., as compared to 250‐500 c.c. blood loss by traditional OPD; (d) short operation time by RPD, as short as 232 minutes. This record of short operation time in RPD was even shorter than the 6‐8 hours taken for traditional OPD; (e) successful RPD in a 95‐year‐old patient, proving RPD is a feasible option in very elderly patients; and (f) low surgical mortality, as low as <3%. Our study showed over 99% of the patients undergoing RPD would like to recommend RPD to those with pancreatic head cancer and periampullary lesions.^2^ The pancreatic team, led by Yi‐Ming Shyr at Taipei Veterans General Hospital, are highly accredited, having received an Award of Symbol of National Quality, Safety, and Quality (SNQ award) for “Minimally Invasive Robotic Pancreatic Surgery ‐ Small Wound for Major Pancreatic Surgery” in Taiwan in 2019.

## 2 MINIMALLY INVASIVE SURGERY IN PANCREATICODUODENECTOMY

Minimally invasive surgery, either laparoscopic or robotic approach, has gained popularity in many surgical fields including pancreatic surgeries.^10^, ^11^, ^12^, ^13^, ^14^ Laparoscopic pancreaticoduodenectomy was introduced early in 1994,^15^ but its application has been limited by its technical complexity and the high level of surgical skill required. Pancreatic reconstruction requires precise placement of suture needles into a small lumen followed by intracorporeal knot‐tying in soft and often friable pancreatic parenchyma. Mastering these complicated operative techniques requires advanced laparoscopic skills and a steep learning curve. With the introduction of da Vinci Robotic Surgical System (Intuitive Surgical, Inc), several limitations related to the laparoscopic approach have been overcome. Providing high‐quality three‐dimensional (3‐D) and optical 10‐15 magnification vision, articulated instruments, greater precision with suture targeting, and elimination of surgeon tremor, robotic approach can even enable complex procedures such as Whipple procedure, which involves extensive and complex resection and reconstruction of pancreas, bile duct, and gastrointestinal tract.^12^, ^16^, ^17^ However, a major concern about the da Vinci Robotic Surgical System is the cost for robotic instruments, one of the reasons that it is not widely accepted as a routine procedure in most centers.^9^, ^10^, ^18^, ^19^, ^20^, ^21^

## 3 TROCHA PORT DESIGN IN ROBOTIC PANCREATICODUODENECTOMY

**FIGURE 2:**
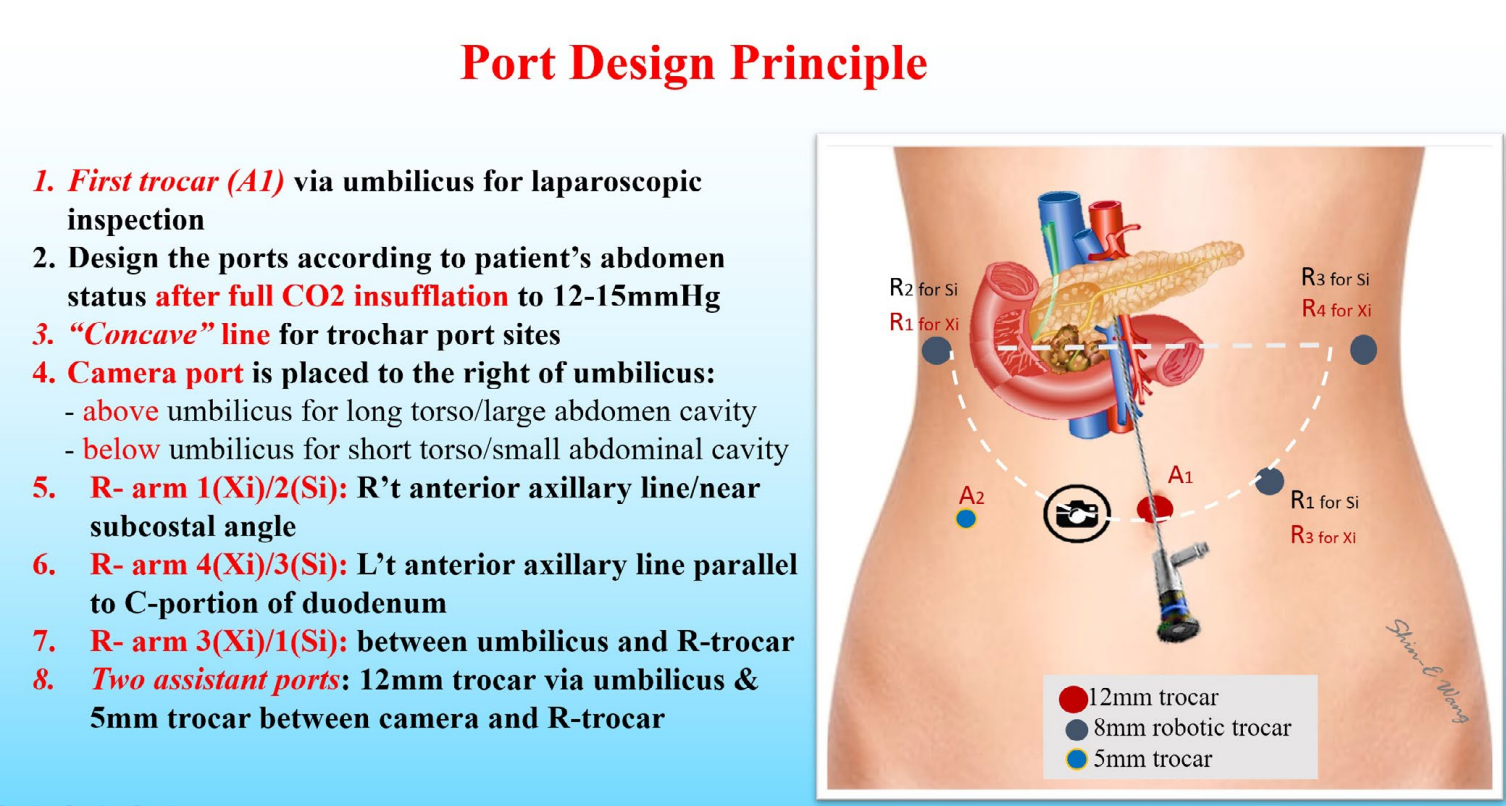
Trocar port design principle with six trocar ports including four for robotic and two for assistant instruments

**FIGURE 3:**
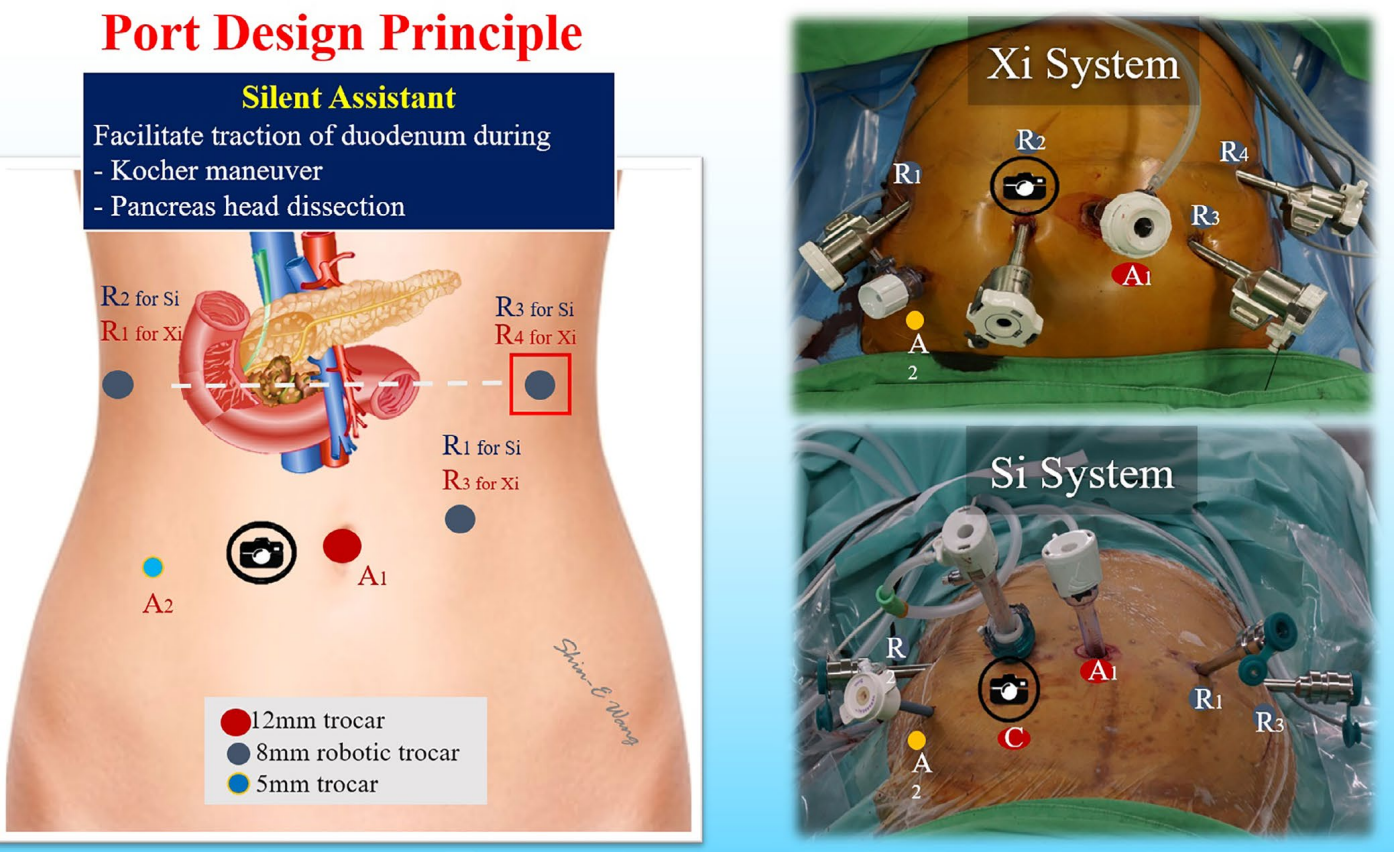
Trocar port design for da Vinci Robotic Surgical Systems

**FIGURE 4:**
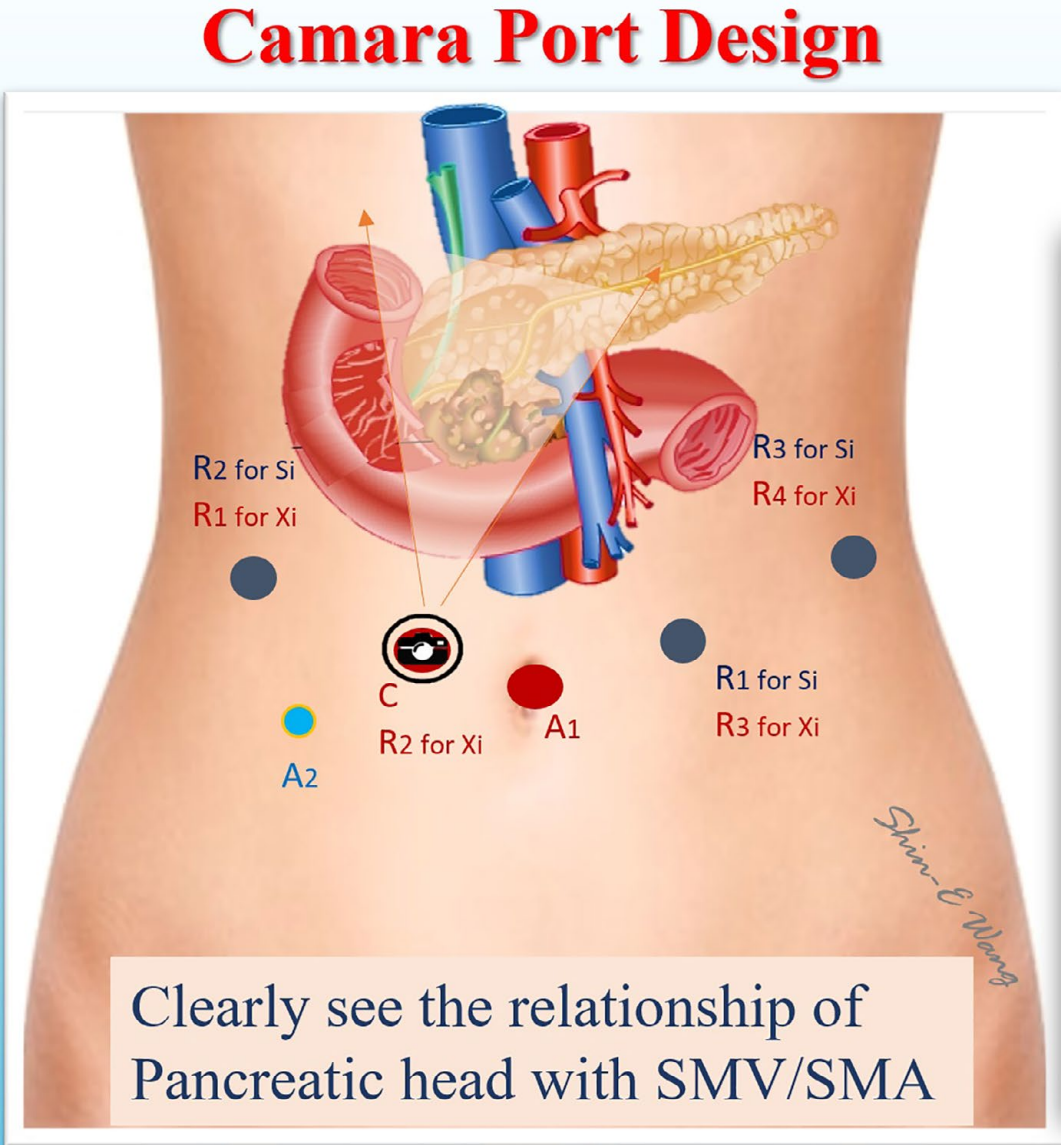
The cameral port indicated with “C” is placed about 5 cm to the right of umbilicus. Be this design, the robotic scope can clearly see the relationship of pancreatic head/uncinated process and superior mesenteric vessels during dissection around these vessels

da Vinci Robotic Surgical System (Intuitive Surgical, Inc) is used to perform RPD. Six ports including four robotic trocars and two assistant ports are used in both da Vinci Si and Xi Robotic Surgical Systems at Taipei Veterans General Hospital (Figure 2).^5^ The trocar designs are similar for da Vinci Si and Xi Surgical Systems (Figure 3). First, a 12‐mm trocha as the assistant port is set up via transumbilical incision, and pneumoperitoneum at a pressure level of 12‐15 mm Hg is established. Laparoscopic inspection is performed first to check the feasibility and resectability of RPD before docking the robotic system. Three 8‐mm robotic ports for working instruments are set up, one along the right anterior axillary line at the same level pancreatic head, another one along the left anterior axillary line at the same level pancreatic head, and the third along the left mid‐clavicular line slightly below the umbilicus level. The 8‐mm Xi or 12‐mm Si camera port is placed at about 3‐5 cm to the right of umbilicus. Thus, the robotic camera scope can clearly see the relationship of pancreatic head and superior mesenteric vessels during pancreatic head dissection (Figure 4). A 5‐mm trocha as an assistant port is usually placed on the right mid‐clavicular line slightly below the camera port.^5^, ^6^

## 4 TECHNIQUE OF PANCREATIC RECONSTRUCTION IN ROBOTIC PANCREATICODUODENECTOMY

Pancreatic reconstruction with a modified Blumgart pancreaticojejunostomy (PJ) has been previously described in detail.^1^, ^5^, ^6^, ^22^ Briefly, three transpancreatic U‐sutures are placed for the horizontal mattress outer sutures on the posterior wall of jejunum, using 3‐0 monofilament synthetic absorbable sutures (PDS™). The U‐sutures are placed at about 0.8‐1 cm from the pancreatic cutting margin. These U‐sutures with needles on them are left untied, kept separate, and held with robotic instruments to facilitate inner layer anastomosis. The inner layer duct‐to‐mucosa anastomosis is performed carefully and precisely by a series of interrupted sutures with 4‐0 absorbable synthetic monofilament suture (MonoPlus®), usually six for a normal pancreatic duct and eight or more sutures for a dilated duct. The inner layer sutures are completed one by one by pair‐watch technique until the last three sutures. After completing the inner layer duct‐to‐mucosa anastomosis, the outer layer horizontal mattress sutures on the anterior wall of jejunum are completed, using previously placed U‐sutures, which are held and organized by the robotic arm.

## 5 MESOPANCREAS DISSECTION

“Mesopancreas dissection,” proposed by Inoue et al,^23^ is used to describe the extent of lymph node dissection during separation of pancreas head‐uncinate process from superior mesenteric vessels. Mesopancreas dissections can be categorized into three levels based on the extent of dissection around and along the superior mesenteric vessels, including level 1 mesopancreas dissection, simply along the right side of superior mesenteric vein (SMV), usually applied for those with benign or low‐malignancy potential; level 2 mesopancreas dissection, along the right side of superior mesenteric artery (SMA), and en bloc resection of the corresponding lymph nodes and mesojejunum, but not including the nerve plexus on the SMA, applied for periampullary cancers; level 3 mesopancreas dissection, including en bloc mesopancreas resection with periadventitial tissues including nerve plexus along the right hemi‐circumference of SMA from 5 to 11 o'clock, just applied for pancreatic head cancer.^8^, ^23^, ^24^, ^25^

## 6 SURGICAL OUTCOMES AFTER ROBOTIC PANCREATICODUODENECTOMY

**FIGURE 5:**
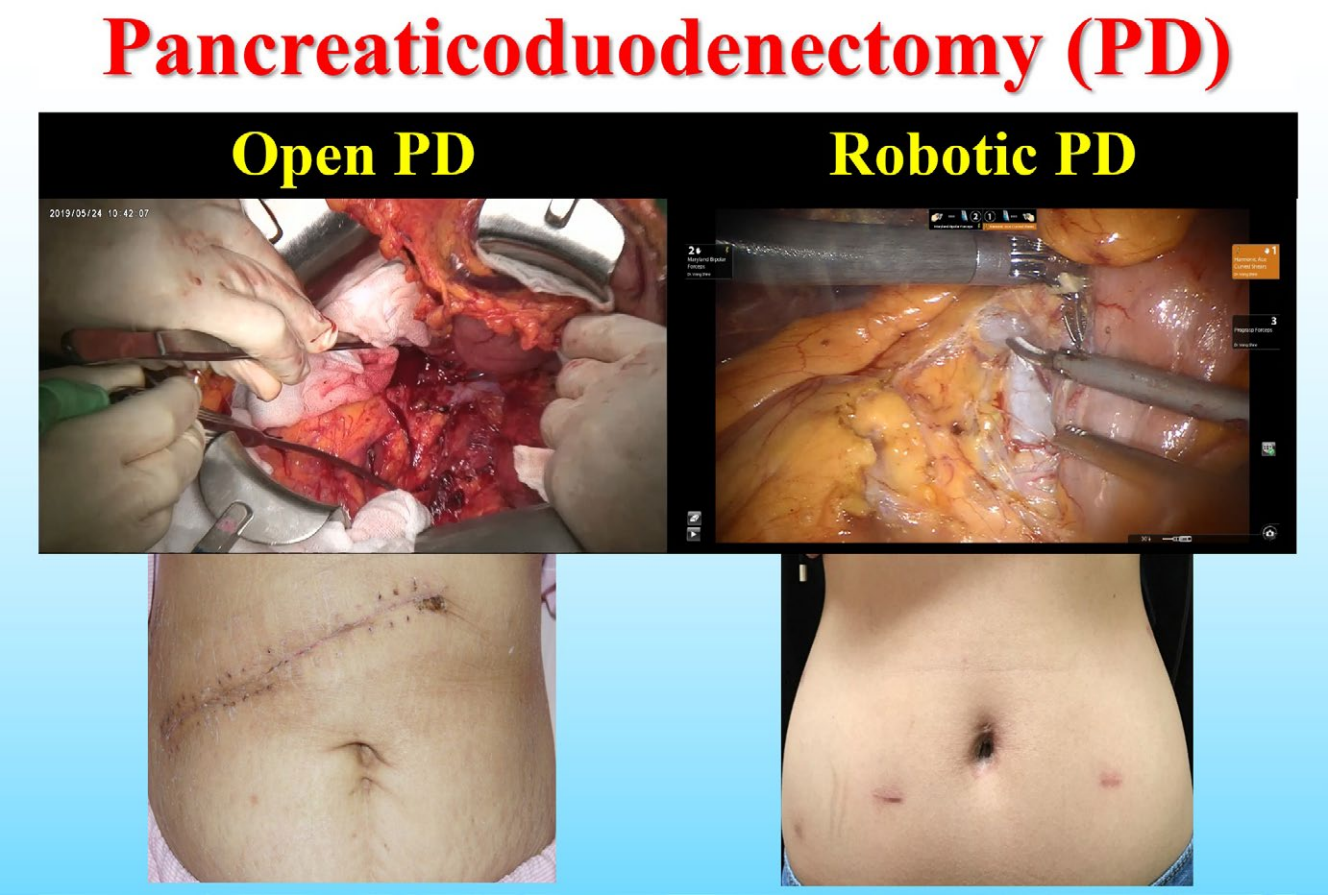
Surgical wounds for robotic and traditional open pancreaticoduodenectomy at Taipei Veterans General Hospital

It has been claimed that RPD has benefits of less delayed gastric emptying, less blood loss, lower wound infection rate, and shorter postoperative hospital stay, as compared with OPD, according to studies and literature reports.^1^, ^5^, ^7^, ^8^, ^9^, ^26^, ^27^, ^28^, ^29^ Our study showed that the biggest complications after RPD are 18.1% occurrence of chyle leakage, followed by 5.7% occurrence of postoperative pancreatic fistula, 4.8% occurrence of intra‐abdominal abscess, 3.8% occurrence of delayed gastric emptying, and post pancreatectomy hemorrhage.^2^ The wounds after RPD and OPD are shown in Figure 5. We conducted a study of patient satisfaction and quality of life using questionnaires for 105 RPD patients. The results revealed that almost all of the patients responded to this RPD‐related survey with “fair” to “excellent” grades for all items, except one (<1%) poor grade for operation service and two (1.9%) “not good” grades for diet tolerance. More than 99% (104/105 = 99.05%) of the patients after RPD were satisfied with the surgical outcomes and would like to recommend RPD to those patients with pancreatic head cancer and periampullary lesions.^2^

## 7 SURVIVAL OUTCOMES AFTER ROBOTIC PANCREATICODUODENECTOMY

**FIGURE 6:**
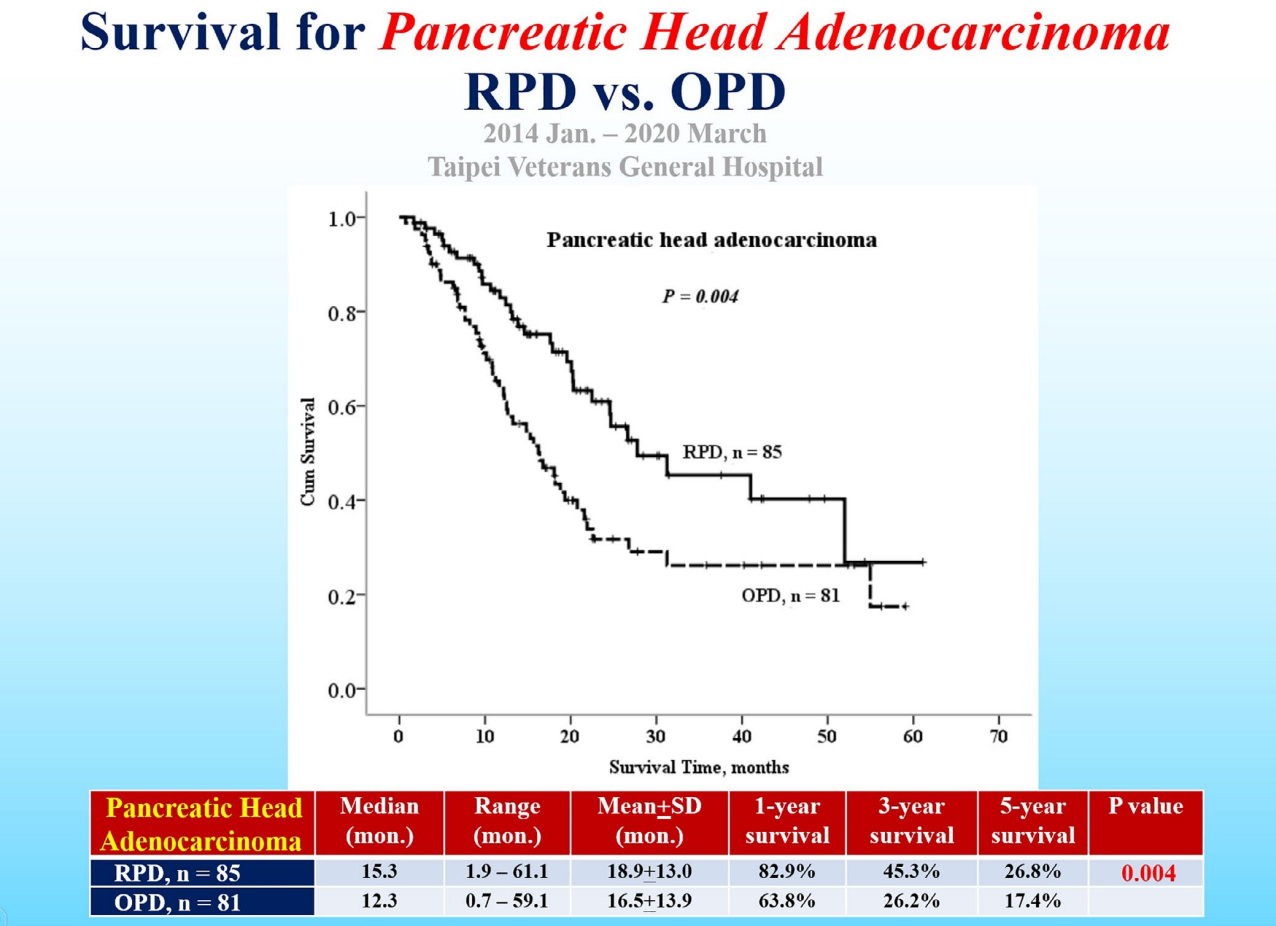
Survival curves for pancreatic head adenocarcinoma after robotic pancreaticoduodenectomy (RPD) and open pancreaticoduodenectomy (OPD)

**FIGURE 7:**
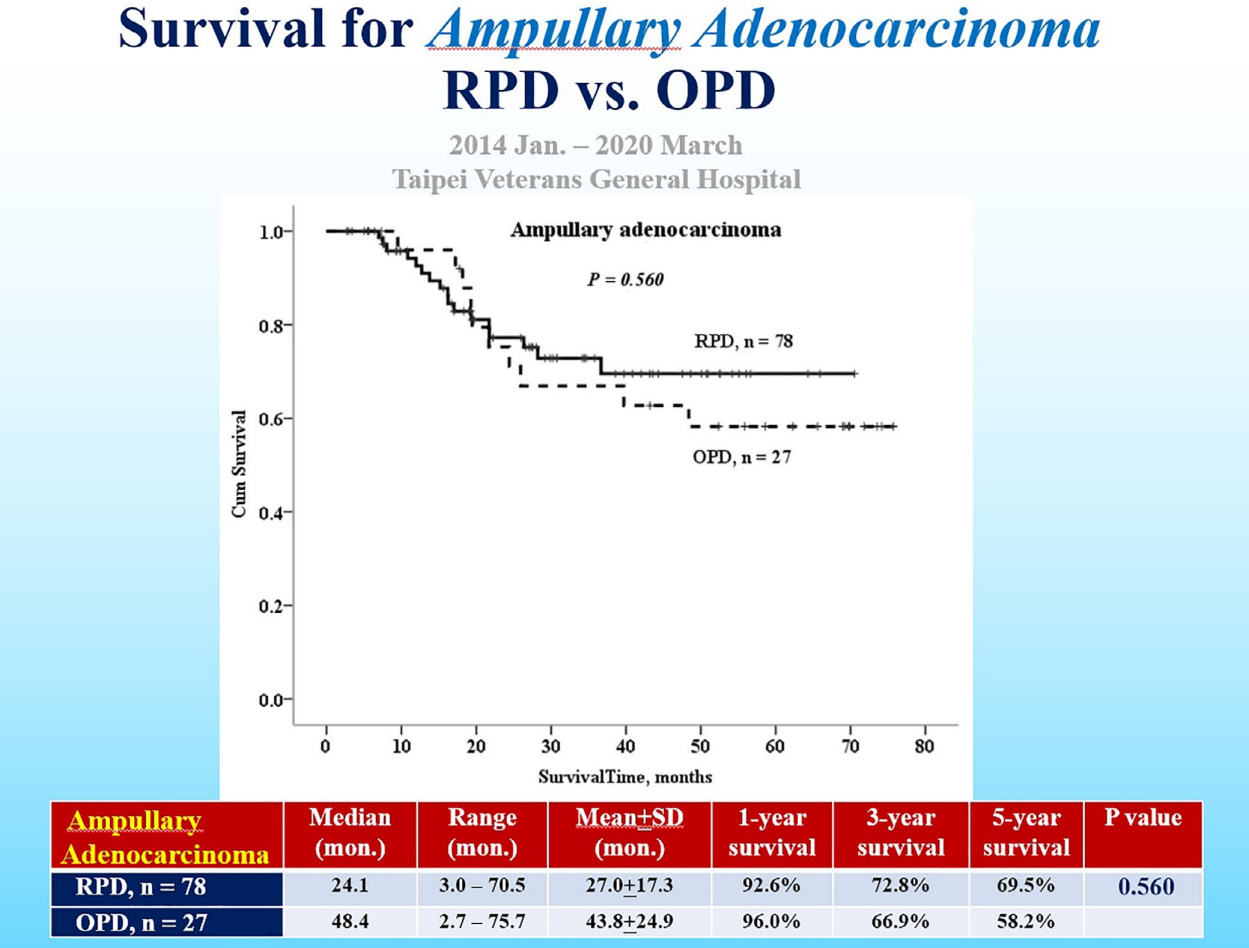
Survival curves for ampullary adenocarcinoma after robotic pancreaticoduodenectomy (RPD) and open pancreaticoduodenectomy (OPD)

Survival outcomes after RPD have not been well‐studied or reported in the literature.^9^ We conducted a retrospective study for survival outcomes of pancreatic head cancer patients undergoing pancreaticoduodenectomy, comparing 85 RPD and 81 OPD patients. This study showed there was a benefit of survival in the RPD group, with 82.9% of 1‐year survival, 45.3% of 3‐year survival, and 26.8% 5‐year survival, as compared with 63.8%, 26.2%, and 17.4%, respectively, in the OPD group, *P* = .004 (Figure 6).^7^ For ampullary cancer, there is no survival difference between RPD and OPD groups (Figure 7).^7^ At least, RPD is not only technically feasible but also oncologically justifiable without compromising the survival outcomes of pancreatic head and ampullary cancers, although selection bias would be inevitable in this retrospective study.^1^, ^2^, ^9^ Prospective randomized control trials or studies of larger sample sizes with long‐term follow‐up are recommended to reach a reliable conclusion.

## DISCLOSURE

Conflict of interest: All authors declare no conflict of interests for this article.
